# The effect of diet on serum 25-hydroxyvitamin D concentrations in dogs

**DOI:** 10.1186/s13104-015-1360-0

**Published:** 2015-09-15

**Authors:** Claire R. Sharp, Kim A. Selting, Randy Ringold

**Affiliations:** Cummings School of Veterinary Medicine, Tufts University, 200 Westboro Road, North Grafton, MA 01536 USA; Veterinary Medical Teaching Hospital, University of Missouri, Columbia, MO 65211 USA; Veterinary Diagnostics Institute, Simi Valley, CA 93063 USA

**Keywords:** Dietary supplement, Salmon oil, Fish oil, Cancer, Biomarker

## Abstract

**Background:**

Vitamin D (vitD) deficiency is linked to many disease states including rickets and cancer, and vitD supplementation to improve response to cancer therapy has been explored. Supplementation may be most appropriate for dogs with suboptimal vitD concentrations. In dogs, the primary source of vitD is diet (predominantly via commercial dog food). Our goal was to determine how food source and supplements affect 25(OH)D concentrations, the storage form of vitD. Serum was collected from clinically healthy dogs, and pet owners were surveyed about food source and supplements. Serum 25(OH)D concentration was measured using a quantitative chemiluminescent assay (LIASON, DiaSorin, Stillwater, MN).

**Results:**

Dogs (n = 320) were tested for serum 25(OH)D concentrations (range 9.5–249.2 ng/mL). Dogs were fed commercial diets from forty different manufactures (n = 292); additionally some dogs were fed homemade diets (n = 18) or a combination of commercial and homemade diets (n = 10). Median serum 25(OH)D concentrations in dogs fed commercial foods ranged from 47.4 to 100.1 ng/mL with an overall median of 67.9 ng/ml (CV 29 %). Analysis for differences among manufacturers was significant (*P* = 0.0006). Serum 25(OH)D concentrations amongst dogs fed homemade diets had the largest range (9.5–129 ng/mL) and the lowest value (9.5 ng/mL). Dogs receiving salmon oil as a supplement (n = 22) had significantly higher serum 25(OH)D (on average a 19.6 ng/mL increase) than those not receiving a supplement (*P* = 0.007).

**Conclusions:**

Serum 25(OH)D concentrations in dogs vary widely which likely reflects varying dietary vitD content. Notable differences exist among manufacturers and brands and may reflect differences in proprietary formulations. Given the variability of measured serum 25(OH)D concentrations in dogs and the importance vitD appears to have on health status, dietary vitD content should be optimized.

**Electronic supplementary material:**

The online version of this article (doi:10.1186/s13104-015-1360-0) contains supplementary material, which is available to authorized users.

## Background

The vitamins D2 and D3 are seco-sterol prohormones whose active metabolite, 1,25-dihydroxyvitamin D [1,25(OH)_2_D], is an important hormone, transcriptional activator, and immunomodulator [1, 2]. Calcitriol [1,25(OH)_2_D] is best known as a key regulator of bone metabolism and calcium homeostasis [1, 2]. Since circulating concentrations of 1,25(OH)_2_D are tightly regulated by the body, calcidiol or serum 25-hydroxyvitamin D [25(OH)D] is the major circulating form of vitamin D and hence best indicator available of vitamin D status in humans and domestic animals [1–3]. In addition to its role in bone health, calcidiol and calcitriol modulate cell growth, neuromuscular and immune function, and reduces inflammation [4, 5]. Many genes, modulated in part by vitamin D, encode proteins that regulate cell proliferation, differentiation, and apoptosis. Many cells have vitamin D receptors, and some convert 25(OH)D to 1,25(OH)_2_D. Expanding models of vitamin D look to its impact on cellular health [4]. “Deficiency”, “insufficiency”, and “sufficiency” are terms that define increasing levels of vitamin D which are linked to many disease states including cancer and other serious diseases [4–6].

Dogs produce negligible amounts of vitamin D via sunlight and rather derive their vitamin D requirement through diet [7]. This is in contrast to humans and other species whose primary source of vitamin D is via sunlight exposure which converts 7-dehydrocholesterol in the skin to vitamin D3 [2, 3]. Since vitamin D is fat soluble, it is stored in adipose tissue. Historically dogs have obtained the vitamin D they need from eating the fat stores of killed prey. However, dogs like humans, experienced a drastic change in the 1900′s as humans moved out of the sunlight into offices and dogs moved to commercial dog food diets. For dogs, the supplementation provided in dog food became their primary source of vitamin D.

Supplementation of commercial dog foods with vitamin D has been adequate to prevent nutritional rickets in dogs [6], however the ideal dose of supplementation and corresponding serum concentrations of vitamin D to maintain cellular health are not known. Normal ranges provided by commercial laboratories for serum concentrations of 25(OH)D in dogs have a wide distribution, and may not reflect ideal serum concentrations. This has been an area of debate in both human [4, 5] and veterinary medicine [6]. Defining vitamin D sufficiency by the range of serum vitamin D concentrations in which concentrations of intact parathyroid hormone plateau may be the most physiologic definition and was the subject of a recent investigation in dogs [6], similar to those conducted in humans [4, 8–10]. Based on this definition in dogs, 25(OH)D sufficiency was defined by serum concentrations of greater than 100 ng/mL [6].

Despite abundant research regarding the role of vitamin D in health and disease in humans, little has been studied in dogs. Recently, new work is discovering a link between low stores of vitamin D and cancer as well as other diseases in dogs. Investigators have found associations of low 25(OH)D with lymphoma, cutaneous mast cell tumors, hyperparathyroidism, kidney disease, inflammatory bowel disease (IBD), cardiovascular disease, and infection [11–16].

The objective of this study was to evaluate the serum vitamin D concentrations in dogs without clinical signs of disease (deemed healthy) to determine how food source and supplements affect circulating blood stores of 25(OH)D.

## Methods

### Cohort

German Shepherd (n = 144), White Shepherd (n = 8), and Golden Retriever dogs (n = 168), recruited from breed clubs across the USA between April 2011 and January 2012, were eligible for inclusion. Dogs were ‘apparently healthy’ according to the owner (and in most cases, veterinarian) with no history of serious disease or current illness. Pet owners completed a questionnaire regarding diet and supplements (Additional file 1). The study was approved by the Clinical Studies Review Committee (Tufts). Institutional Animal Care and Use Committee approval was not required by the University of Missouri at the time of this study. Additionally each owner was required to provide signed consent. The majority of the dogs enrolled in this study were also enrolled into the control group of a previously published study investigating vitamin D status and cancer risk [6].

### Sample collection

Blood was collected and centrifuged within 1 h of sample collection and a minimum of 0.5 mL serum was harvested. The serum was placed in an airtight, freezer-resistant plastic tube and stored at −20 °C or colder. Tubes were coded so that the identity of the sample was known only to the principle investigator; the laboratory was blinded. Samples were stored frozen and shipped on ice to a commercial laboratory for subsequent batch analysis.^1^

### 25(OH)D assay

Serum 25-hydroxyvitamin D [25(OH)D] was measured at a commercial laboratory^1^ using the LIASON^2^ 25(OH)D assay, a direct, competitive chemiluminesence immunoassay (CLIA) for the quantitative determination of 25(OH)D in serum. Intra and inter-assay precision were 4.1 and 3.4 % respectively. This method has been previously validated and reported [6].

### Statistical analysis

Statistical analysis was performed using commercially available software.^3^ A Kruskal–Wallis 1-way analysis of variance was used to compare serum 25(OH)D concentrations among dogs fed different brands of dog food. Nonparametric data are reported as median [range]. Additionally values representing the middle values in the first and third quartiles (Q1 and Q3 respectively) are reported. Based on a previously published report, vitamin D sufficiency was classified as 25(OH) of >100 ng/mL, vitamin D insufficiency as 25–100 ng/mL, and vitamin D deficiency as <25 ng/mL [6].

## Results

### Study population

**Fig. 1:**
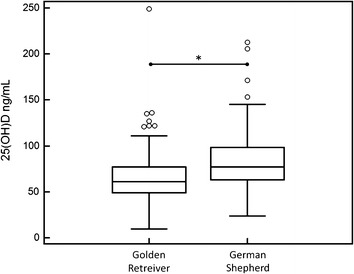
*Box* and *whiskers plot* of serum 25(OH)D concentrations in dogs by breed. *Open circles* represent outliers that are more than 2.5 standard deviations from the group. Significantly different groups are represented by *asterisk*

A total of 320 dogs met the criteria and were subsequently enrolled. Signalment information is listed in Table 1. There was no significant difference in serum 25(OH)D concentrations by age however there was a significant difference by breed (Fig. 1). German Shepherd dogs had a significantly higher serum 25(OH)D concentrations than Golden Retrievers (76.5 ng/mL [23.9–212.8], 60.6 ng/mL [9.5–249.2], respectively, *P* < 0.0001).

**Table 1:** Comparison of age, sex, and breed in healthy dogs whose serum 25(OH)D concentrations were evaluated

Signalment information	
N	320
Age median years (range)	7 (0.4–14.5)
Sex	F (74), FS (108), M (77), MN (61)
Breed	German Shepherd (144)
	Golden Retreiver (168)
	White Shepherd (8)

*F* sexually intact female, *FS* spayed female, *M* sexually intact male, *MN* neutered male

### Comparison of 25(OH)D concentrations

#### Commercial diet manufacturers and brands

**Fig. 2:**
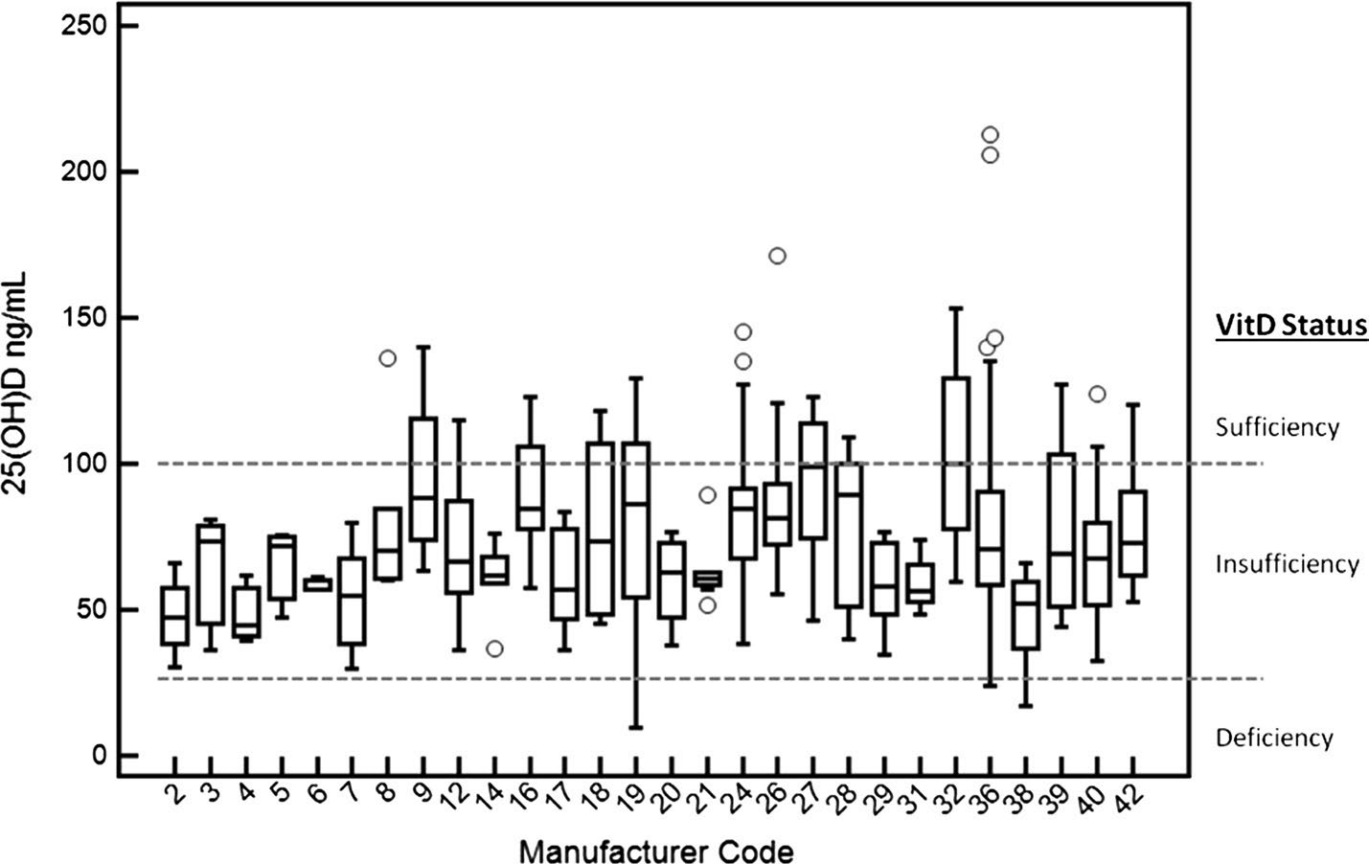
*Box* and *whiskers plot* of serum 25(OH)D concentrations in dogs by brand/manufacturer of the commercial dog food that they are fed, and including homemade diets (code 19). Only those with three or more subjects are presented. See Table 2 for manufacturer code

Dogs were fed commercial dog foods from 40 different brands. In total, 292 dogs were fed commercial dog food only, 18 dogs were fed a homemade diet only, and ten dogs received a combination of commercial and homemade food. See Fig. 2 and Table 2 for details. Overall serum 25(OH)D concentrations ranged from 9.5 to 249.2 ng/mL, with median, Q1, Q3 at 69.7, 54.5, 88.1 ng/mL, respectively. Dogs consuming commercial dog food only had serum 25(OH)D concentrations that ranged from 16.9 to 249.2 ng/mL with median, Q1, Q3 at 67.9, 54.2, 85.3 ng/mL respectively. Dogs consuming a homemade diet only had the most variation (excluding outliers) in their serum concentrations of 25(OH)D, and the lowest value, ranging from 9.5 to 129.0 ng/mL. Group median serum 25(OH)D concentrations in dogs fed homemade diets were significantly different than those in dogs fed some particular brands of commercial foods (data not shown), however when comparing all dogs fed commercial diets to all dogs fed homemade diets there was no difference in serum 25(OH)D concentrations.

**Table 2:** Serum 25(OH)D concentrations (ng/mL) in dogs classified according to the brand/manufacturer of the commercial dog food they are fed

Manufacturer	Code	Cohort	Min	Max	Median
Acana		2	38.3	249.2	
Blue buffalo	2	4	30.4	65.9	47.4
Bravo	3	3	36.0	80.7	73.3
Breeder’s choice	4	3	39.5	61.9	44.9
Canidae	5	7	47.5	75.5	71.9
Canine Caviar	6	3	56.8	61.1	57.1
Diamond	7	15	29.9	79.9	54.7
Dick Van Patten	8	6	60.3	136.0	70.1
Dr. Gary’s	9	4	63.2	140.0	88.1
Eagle pack		1	101.0	101.0	
Earthborn holistic		1	107.0	107.0	
Eukanuba	12	13	36.2	115.0	66.3
Evanger’s		2	40.5	56.4	
Exceed	14	6	36.5	76.1	61.9
Exclusive		2	69.8	95.6	
Fromm family	16	12	57.2	123.0	84.4
Hill’s	17	5	36.1	75.7	56.9
Holistic select	18	4	45.4	118.0	73.5
Homemade diet	19	18	9.5	129.0	86.2
Honest kitchen	20	4	37.8	76.4	63.0
Iams	21	8	51.5	89.2	60.5
Joe and Jack’s		1	73.2	73.2	
K-9 kravings		1	75.4	75.4	
Kirkland	24	26	38.4	145.0	84.4
Life’s abundance		1	61.8	61.8	
Mixed	26	10	55.3	171.2	81.6
Natura	27	9	46.4	123.0	98.9
Nature’s variety	28	10	40.1	109.0	89.2
Nutri source	29	5	34.6	76.8	57.8
Nutrisca		1	122.0	122.0	
Nutro	31	9	48.2	74.1	56.2
Orijen	32	4	59.7	153.2	100.1
Pedigree		1	74.1	74.1	
Premium edge		1	73.1	73.1	
Pro pac		1	75.3	75.3	
Purina	36	53	23.9	212.8	70.8
Red flannel		1	47.0	47.0	
Royal canin	38	6	16.9	66.1	52.1
Solid gold	39	4	43.9	127.0	69.1
Taste of the wild	40	44	32.6	124.0	67.7
VeRUS		2	44.8	45.0	
Wellness	42	7	52.7	120.0	72.9

Code represents the manufacturers code and correlates with Fig. 2. Cohort refers to the number of dogs in each diet group. Min, Max and Median refer to serum 25(OH) concentrations

### Supplements

**Fig. 3:**
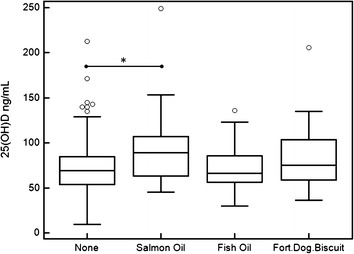
*Box* and *whiskers plot* of serum 25(OH)D concentrations in dogs by dietary supplementation status. 214 dogs did not receive a supplement. The supplements used were salmon oil (n = 22), fish oil (n = 61), and fortified dog biscuits (n = 23). *Open circles* represent outliers that are more than 2.5 standard deviations from the group. Significantly different groups are represented by *asterisk*

Approximately one-third of dogs in this study were fed some sort of dietary supplement that contained vitamin D on a regular basis (Fig. 3). The supplements used were salmon oil (n = 22), fish oil (n = 61), and fortified dog biscuits (n = 23). The median serum 25(OH)D concentrations for dogs not receiving a supplement, and those receiving salmon oil, fish oil, and fortified dog biscuit, were 69.4, 89.0, 66.1, 75.0 ng/mL, respectively. There was no significant difference in the serum 25(OH)D concentrations between dogs not receiving a supplement, and those being supplemented with either fish oil or fortified dog biscuit. Dogs receiving salmon oil had significantly higher serum 25(OH) concentrations than dogs that did not receive a dietary supplement (89.0 ng/mL [45.4–249.2], 69.4 ng/mL [9.5–212.8], respectively, *P* = 0.007).

### Sex and intact status

**Fig. 4:**
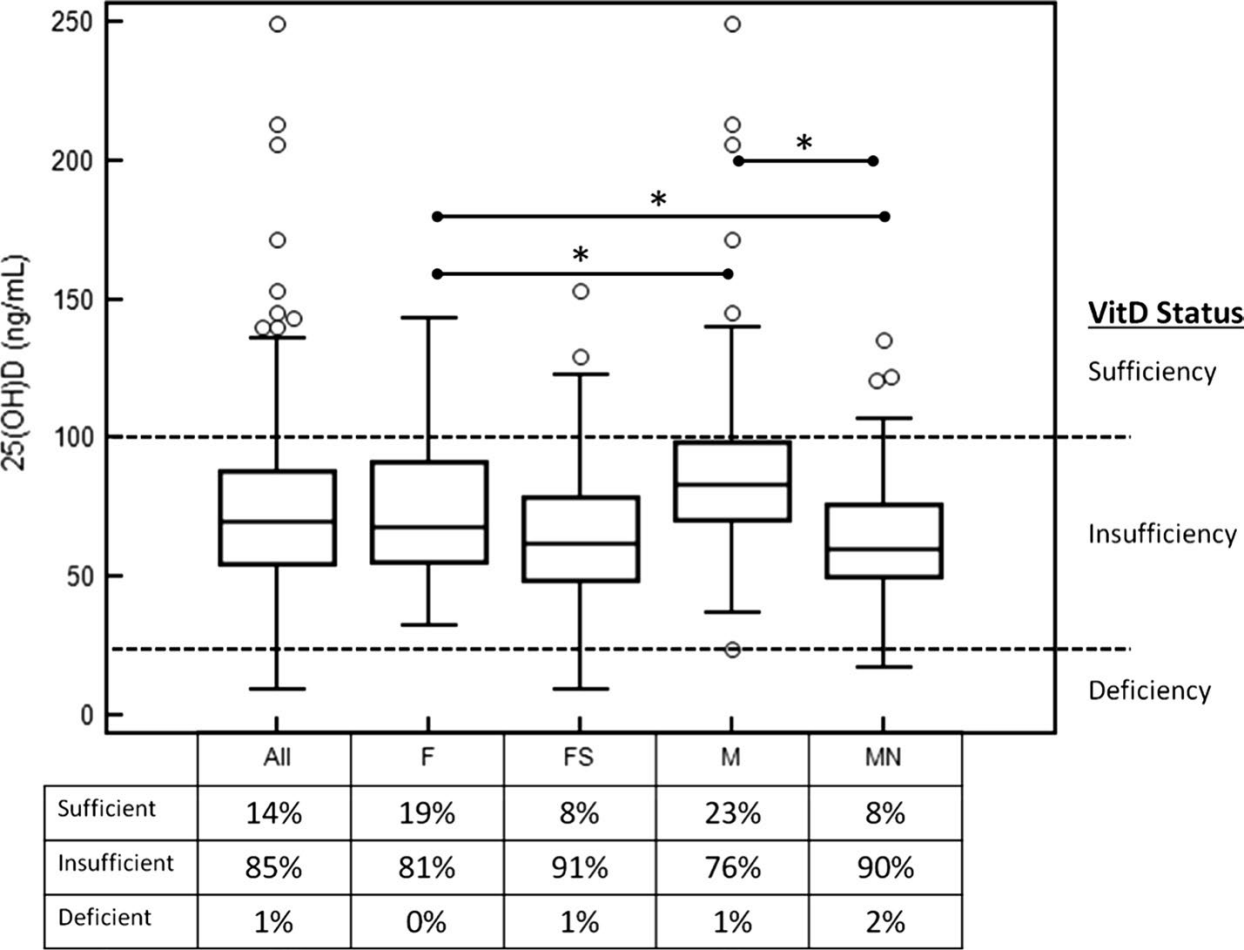
*Box* and *whiskers plot* of serum 25(OH)D concentrations in dogs by sex and intact status. *Open circles* represent outliers that are more than 2.5 standard deviations from the group. Significantly different groups are represented by *asterisk*

As shown in Fig. 4, sexually intact male dogs had significantly higher serum 25(OH)D concentrations than sexually intact female dogs (83.3 ng/mL [23.9–249.2]), 67.7 ng/mL [32.3–143.0], respectively, *P* = 0.001). Sexually intact male dogs also had significantly higher serum 25(OH) concentrations than neutered male dogs (83.3 ng/mL [23.9–249.2], 60.4 ng/mL [16.9–135.0], respectively, *P* < 0.0001). Sexually intact female dogs appeared to have slightly higher serum 25(OH)D concentrations (than spayed females (67.7 ng/mL [32.3–143.0], 61.8 ng/mL [9.5–153.2], respectively), however this did not reach statistical significance (*P* = 0.06). There was no significant difference in the serum 25(OH)D concentrations between neutered male and spayed female dogs (median 60.4 ng/mL [16.9–135.0], 61.8 ng/mL [9.5–153.2], respectively, *P* = 0.826).

## Discussion

Serum 25(OH)D concentrations vary widely among ‘apparently healthy’ dogs (9.5–249 ng/mL). Because skin synthesis of vitamin D is minimal, these differences can be attributed to dietary intake or absorption. Breed had a significant influence on serum vitamin D concentrations with German Shepherds showing a 26 % higher median concentration than Golden Retrievers. This difference suggests that intestinal absorption of vitamin D may vary by breed.

Salmon oil supplementation, known for its high concentration of vitamin D, has a significant impact on serum vitamin D concentrations, whereas the effect of fortified dog biscuits was far less. Surprisingly, fish oil supplementation had no effect, though information on dose or timing relative to meals was not available. Differences in bio-availability and rate of absorption may affect how much vitamin D is actually absorbed, especially given that vitamin D is fat soluble. Unfortunately the exact vitamin D content of specific diets and supplements is not routinely reported on the product and thus correlations between the diet vitamin D content and serum vitamin D concentration in the dogs are not possible.

In this study sex and intact versus spay/neuter status affected serum vitamin D concentrations. Median 25(OH)D was 9 % lower in spayed compared to intact females, but 27 % lower in neutered compared to intact males. Additionally, intact male dogs had significantly higher 25(OH)D concentrations than intact female dogs. However, there was no significant difference in 25(OH)D concentrations between neutered males and spayed females. This suggests that sex hormones affect vitamin D absorption and that male hormones have a bigger influence on vitamin D absorption than female hormones. An alternative hypothesis is that spay/neuter status may affect the quantity of food eaten and thus indirectly affect vitamin D intake (i.e. sexually intact male dogs may eat more than neutered males or females, thereby resulting in higher serum vitamin D concentrations). These factors could have been more thoroughly explored by determining the quantity of food eaten (as well as the vitamin D content of the food), however such investigations were outside of the scope of the present study.

An evolving model of vitamin D and its impact on health is redefining levels needed to reduce the risk for cancer and other serious diseases. Vitamin D deficiency, below the level needed for proper skeletal development, is typically defined as less than 20–25 ng/mL [3]. In dogs, low 25(OH)D has been associated with lymphoma, mast cell neoplasia, sarcoma, heart and kidney disease, hemangiosarcoma, infection, and IBD [6, 11–15]. Recent data has defined vitamin D sufficiency as serum concentrations at or above 100 ng/mL [6]. In the cohort reported in this study, and using these limits, many apparently healthy dogs are vitamin D insufficient and a few are even deficient.

This study has some limitations that should be considered. Firstly, dogs were considered apparently healthy based on lack of clinical signs or physical examination abnormalities suggestive of disease. It is certainly possible that some dogs in this study had occult disease that influenced the measured serum 25(OH)D concentrations. While the total number of dogs enrolled in this study was reasonable, the numbers of dogs receiving different dietary supplements was considerably lower, and thus a type II error associated with analysis of a link between dietary supplements and 25(OH) concentrations is possible. Additionally, dogs enrolled in this study represented a limited number of dog breeds. It is currently unknown whether breed affects vitamin D status in dogs. Furthermore, although it can be assumed that differences in serum 25(OH)D are due, at least in part, to different dietary content of vitamin D, dietary vitamin D content was not analyzed as part of this study.

## Conclusions

This study showed that serum 25(OH)D concentrations vary widely in dogs, likely as a consequence of dietary vitamin D intake. Dogs supplemented with salmon oil had significantly higher serum 25(OH)D than dogs not receiving a dietary supplement, or those receiving fish oil or fortified dog biscuits. In this study intact male dogs had significantly higher serum 25(OH)D concentrations than intact females, and neutered male dogs. Given the wide distribution of measured 25(OH)D and the importance vitamin D appears to have on health status, vitamin D supplementation should be optimized.

## Additional file

**Additional file 1.** Diet questionnaire.

## Abbreviations

25(OH)D: 25hydroxyvitaminD
CLIA: chemiluminescence immunoassay
IBD: inflammatory bowel disease
VDI: Veterinary Diagnostics Institute

## Footnotes

1 Veterinary Diagnostics Institute, Simi Valley, CA, USA.

2 DiaSorin Inc., Stillwater, MN, USA.

3 MedCalc Software, version 12.7, Belgium.
