# Supplementary figures and images for: The effect of diet on serum 25-hydroxyvitamin D concentrations in dogs
Source: BMC Res Notes. 2015 Sep 15;8:442. doi: 10.1186/s13104-015-1360-0 (PMC4570747; doi:10.1186/s13104-015-1360-0)

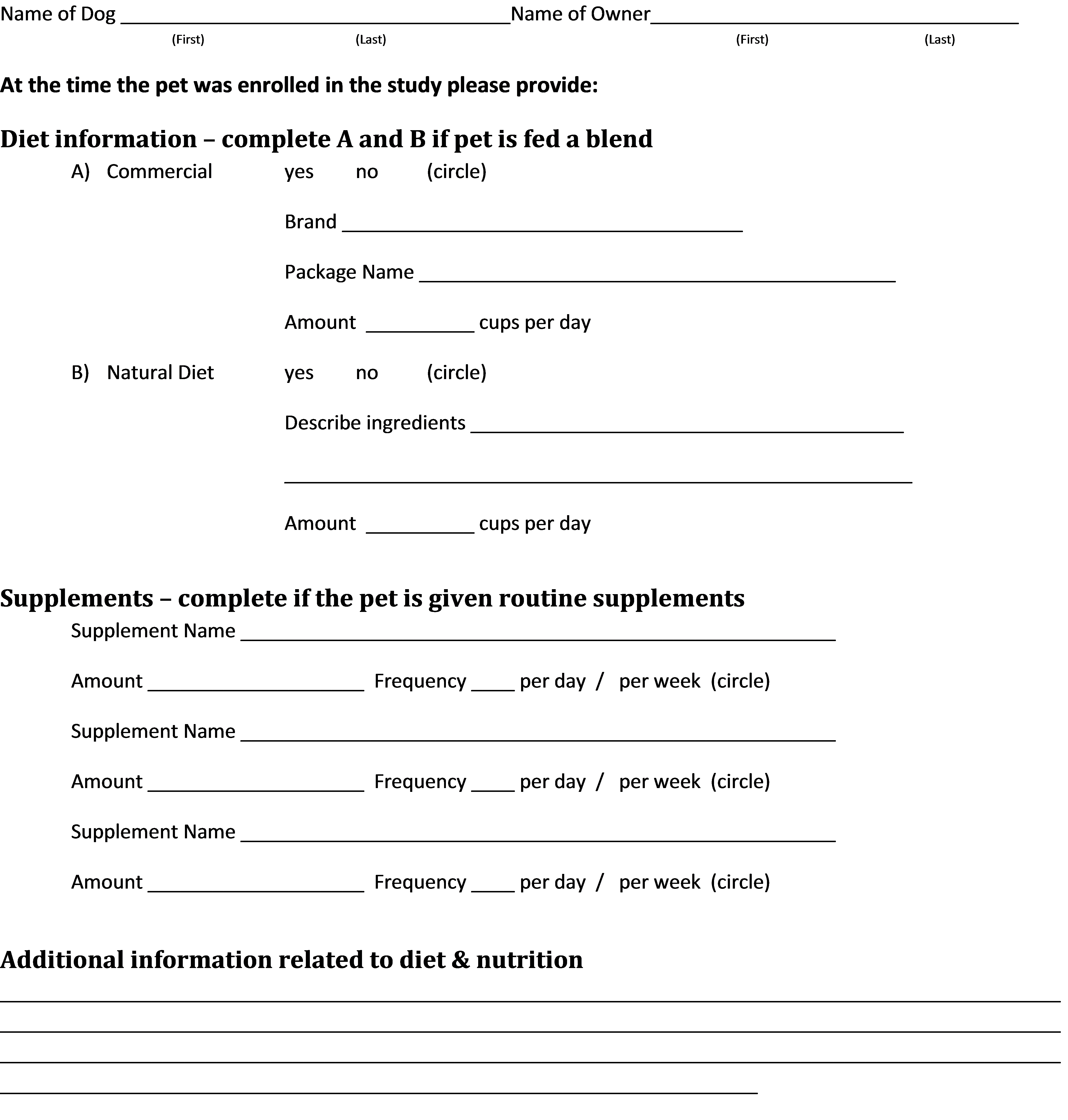

Supplement: Supplementary file 1 — Additional file 1. Diet questionnaire. [file 13104_2015_1360_MOESM1_ESM.png]
